# Raman Spectroscopy Unfolds the Fate and Transformation of SWCNTs after Abrasive Wear of Epoxy Floor Coatings

**DOI:** 10.3390/nano14010120

**Published:** 2024-01-03

**Authors:** Amaia Soto Beobide, Rudolf Bieri, Zoltán Szakács, Kevin Sparwasser, Ioanna G. Kaitsa, Ilias Georgiopoulos, Konstantinos S. Andrikopoulos, Gunther Van Kerckhove, George A. Voyiatzis

**Affiliations:** 1 Foundation for Research and Technology-Hellas (FORTH), Institute of Chemical Engineering Sciences (ICE-HT), Stadiou Str., 265 04 Rio-Patras, Greece; candrik@iceht.forth.gr (K.S.A.); gvog@iceht.forth.gr (G.A.V.); 2 Stat Peel Ltd., Stampfgasse 4, CH-8750 Glarus, Switzerland; rudolf.bieri@statpeel.com (R.B.); zoltan.szakacs@statpeel.com (Z.S.); 3 Department of Physics, University of Patras, 265 04 Rio-Patras, Greece; ioanna.kaitsa@protonmail.com; 4 MIRTEC S.A., Thiva Branch, 76th km of Athens-Lamia National Road, 320 09 Schimatari, Greece; i.georgiopoulos@mirtec.gr; 5 OCSiAl Europe Sarl, Rue de la Poudrerie 1, 3364 Leudelange, Luxembourg; gunther.van.kerckhove@ocsial.com

**Keywords:** single-wall carbon nanotubes, release, tribology, epoxy, fate, Raman spectroscopy

## Abstract

Nanomaterials are integrated within consumer products to enhance specific properties of interest. Their release throughout the lifecycle of nano-enabled products raises concerns; specifically, mechanical strains can lead to the generation of fragmented materials containing nanomaterials. We investigated the potential release of single-walled carbon nanotubes (SWCNTs—brand TUBALL™) from epoxy composite materials. A pin-on-disk-type tribometer was used for the accelerated mechanical aging of the nanocomposites. A pristine nanocomposite material, abraded material and debris obtained from the abrasion in the tribometer were analyzed by Raman spectroscopy. The airborne-produced particles were captured using particle collectors. Stat Peel’s Identifier C2 system was used to monitor the SWCNT content of respirable particles produced during the abrasion test. The SWCNT amounts found were below the LoQ. The Raman spectra conducted on the Stat Peel filters helped identify the presence of free SWCNTs released from the epoxy matrix, although they were notably scarce. Raman spectroscopy has been proved to be a crucial technique for the identification, characterization and assessment of structural changes and degradation in SWCNTs that occurred during the abrasion experiments.

## 1. Introduction

Nanoscience and nanotechnology are exciting and rapidly evolving fields that deal with the manipulation and application of materials consisting of solid particles at the nanoscale. These fields have the potential to revolutionize various industries and bring about numerous benefits; as novel technologies and efficient materials, they could have a profound impact on healthcare with applications in drug delivery, diagnostics, and medical and energy-efficient processes, among many others. According to the recently updated European Commission recommendation on the definition of nanomaterial (NM) for legislative purposes, ‘nanomaterial’ means a natural, incidental or manufactured material consisting of solid particles, where 50% or more of these particles in the number-based size distribution typically range from 1 to 100 nanometers in sizes in one or more of their external dimensions [1]. Additionally, nanomaterials are the particles that have an elongated shape, such as a rod, fiber or tube, where two external dimensions are smaller than 1 nm and the other dimension is larger than 100 nm, like the SWCNTs. In case nanomaterials are used amongst other ingredients in a formulation, the entire product will not become a nanomaterial. In this context, advanced multicomponent nanostructured materials may provide superior functional properties, but they can also exhibit an increased toxicological risk, and the environmental fate of the involved nanomaterials should be further studied within the context of human and environmental health [2,3].

It is generally acknowledged that the mechanical, thermal, and electrical properties of polymers can be greatly enhanced by the incorporation of carbon-based nanoparticles in their matrix [4,5]. To this end, composites have been proposed in various applications, several of which have already turned into commercialized products [6]. Among the carbon-based nanomaterials, carbon nanotubes (CNTs) are the ones that cover many, if not most, of these applications. Categorized as nanomaterials, there have been extensive studies on their toxicological effects on humans as well as on the environment [7]. Nevertheless, the results are not yet straightforward, even though there are studies that claim that several parameters such as the type of CNTs, as well as their length, aspect ratio, surface area, degree of aggregation, purity, concentration, and dose, may affect their toxicity [8]. It is generally understood that although there exist diverse toxicological studies which concern mainly pristine CNTs, the evaluation and justification of their toxicity in realistic cases requires specific studies aimed at the release of CNTs from the corresponding nanocomposites during their use and lifecycle [9]. Furthermore, it is of considerable importance to understand the process of carbon-based nanomaterials’ transformation and/or biodegradation, which may facilitate their lifetime regulation [10]. The release pathways of CNTs into the natural environment during the polymer nanocomposite lifecycle along with the techniques introduced for their identification/quantification in environmentally relevant matrices were extensively discussed in the critical review article of E.J. Petersen et al. [11]. That same year, L.G. Cena a and T.M. Peters [12] reported a study on airborne particles emitted during the handling of MWCNTs as well as after the mechanical processing of their epoxy nanocomposites. They demonstrated that the nanocomposites, after being sanded, generate particles in the nanometer scale with protruding CNTs; they pointed out that the toxicity of these particles is unknown. Schlagenhauf et al. [13,14] published a work on the release of CNTs from an epoxy–MWCNTs nanocomposite during an abrasive wear process. They claimed a detection of free-standing individual MWCNTs and suggested precautions when handling materials of unknown toxicity. The effects of sander speed and sandpaper grit (determining the abrasion energy) on the airborne particle emission from CNT-containing materials have been also studied [15], and no free CNTs were observed apart from tests involving 4% CNT test sticks. Wiesner et al. attempted to study the release of MWCNTs from polymeric matrices after controlled mechanical action [16]. They stated that the MWCNTs were largely encapsulated in the polymeric matrix of the abraded fragments and that although the possibility for MWCNTs to be free from nanocomposites appears to be low, further investigation on the potential release of free MWCNTs from abraded plastic materials is required. More recently, the carbon nanoparticles’ structural degradation after friction testing making use of ball-on-disc friction tests was characterized by Raman spectroscopy [17]. Raman analysis revealed a direct correlation between pattern depth, lubricity, and CNT degradation.

The necessity of new and sophisticated methods to measure MWCNT exposures in realistic release scenarios has already been addressed [18]. Finally, protocols concerning the simulation of mechanical ageing of the composites as well as the sampling and characterization procedures should be properly defined.

To recapitulate, when a final product containing nanoparticles is used, it is possible that those particles could become airborne due to wrinkling, folding, breaking, drilling, cutting, etc. It is important to know whether during the normal use of the product, the release of inhalable nanoparticles can occur, causing a possible exposure scenario for workers or consumers. Taking into account the number of parameters involved, the study of CNTs’ release from their composites after mechanical processes is a challenging task.

The aim of the current work is to study the release of CNTs from a commercial antistatic flooring material in which SWCNTs are embedded in epoxy resin. A pin-on-disk-type tribometer was used for the accelerated mechanical aging of the nanocomposites while sampling of the release fragments was achieved by the utilization of three different collection procedures (none of which included immersion followed by shaking/sonication): (i) macroscopically debris collected near the tester; their maximum dimensions being a few hundreds of microns, (ii) particles (maximum dimensions of few microns) collected from five distinct filtration slides from a Stat Peel’s Identifier detection system placed inside the tribometer chamber used to isolate the sliding surfaces during the abrasion test as well as outside the tribometer chamber (approximately 2 m away), and (iii) particles (maximum dimensions of few microns) collected from an Apex2 Casella particle collector sampling aerosol particles from inside the tribometer box. Scanning electron microscopy identified the morphology of the released particles observed on the surface of the filters used for their entrapment, while Raman spectroscopy enabled the identification and characterization of SWCNTs. Raman spectroscopy is a particularly significant technique for the analysis of carbon-based materials [19,20]. It probes the vibrational modes, and hence the chemical structure of molecules, providing a unique structural signature (spectrum) that can be used to identify the structure of materials at molecular level. One of its advantages is that it requires minimum/no sample preparation which might alter or damage the sample. Raman spectroscopy was able to characterize the concentration, the dispersion of the SWCNTs in the composite material, and released fragments as well as identify structural alterations at the molecular level imposed by the mechanical treatment. Characterization involved (i) samples freshly prepared, (ii) the same samples after the accelerated mechanical aging, as well as (iii) isolated macroscopically observed fragments that were collected during the accelerated aging. It is shown that both the concentration and dispersion of SWCNTs differ in each type of sample. Raman spectroscopy provides reliable information about free CNT content and its degradation; this information is valuable for industries and risk assessors as it aids in evaluating the exposure potential of CNT-based products. 

## 2. Materials and Methods

### 2.1. Materials

TUBALL™ SWCNTs (single-walled carbon nanotubes brand name with outer diameter 1.6 ± 0.4 nm, length > 5 μm) and TUBALL™ Matrix 207 were provided by OCSiAl- Europe Sarl (Luxembourg). TUBALL™ MATRIX 207 is a concentrate based on TUBALL™ single-wall carbon nanotubes specifically designed to provide superior electrical conductivity to solvent-free epoxy systems while retaining mechanical properties and minimally impacting the host matrix; it is a mix of Araldite (Oxirane, mono (C12-C14-alkyoxy methyl derivatives) and SWCNTs in a proportion of 90/10.

**Figure 1:**
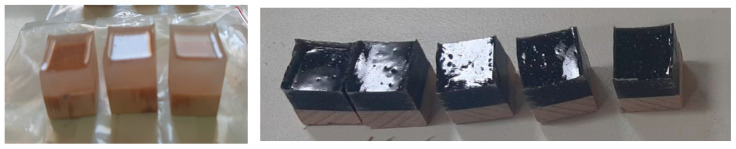
Images of samples for abrasive wear: epoxy reference (**left**) and epoxy coated with TUBALL™ Matrix 207 (**right**). Samples were applied on wood.

For the preparation of specimens for abrasive wear tests, the epoxy resin used was DER 351 (Olin Corporation, Clayton, MO, USA), which is a C12–C14 aliphatic glycidyl ether modified bisphenol A/F based epoxy resin of low viscosity. The used curing agent DEH 488 (Olin Corporation, Clayton, MO, USA) is a low-viscosity, modified cycloaliphatic polyamine-based curing agent for epoxy. The resin hardener ratio was ratio was 100:34.71. The nanocomposite was prepared in a 2-step dilution process. The TUBALL™ SWCNTs were pre-dispersed in a ratio 2 wt.% TUBALL™ MATRIX 207:98 wt.% DER 351, with a high-speed dissolver and a cowl blade mixer for 20 min, at a mixing speed of 10 m/s. This Pre-mix was diluted to a final loading of 0.1 wt.% of TUBALL™ MATRIX 207 to the full system and mixed for 5 min more at 10 m/s. A degassing step was performed in a vacuum oven, which was subsequently followed by the addition of the curing agent, which was mixed for 3 min at 4 m/s. The samples were cast on wooden substrates and cured for 24 h at ambient temperatures (the final thickness of the epoxy resin was ~1 cm), samples are shown in Figure 1. 

The weight fraction of SWCNTs in the nanocomposite is 0.01 wt.%. This is because the dispersion of 0.1% TUBALL™ Matrix 207, which is a mixture of Araldite and SWCNTs in a 90/10 weight ratio, was incorporated into the epoxy.

### 2.2. Abrasion and Particle Collection

**Figure 2:**
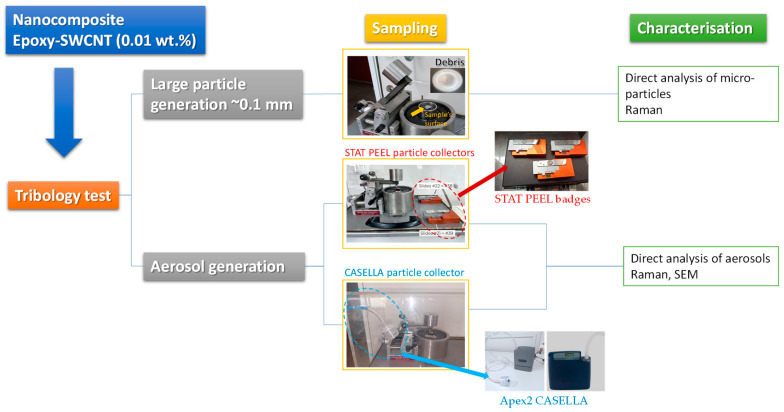
Schematic representation of experimental setup used for the (nano)particle release collection from epoxy–TUBALL M207 composites submitted to abrasion.

Tribology tests were used to simulate different wear mechanisms that are imposed during real-life use in the epoxy coated with SWCNT samples and to evaluate the potential release of particles. The abrasive wear test was performed on a CSEM pin-on-disc tribometer following the ASTM G99-05 procedure. During pin-on-disc experiments, the applied load was 1 N, while stainless steel balls of 6 mm in diameter were used as counter bodies; the specimens had a rotational speed of 50 rpm. The number of cycles was as many as necessary to develop deep wear tracks on each specimen surface and consequently create abrasion debris for further analysis and investigation. Three different approaches were followed to collect particles from potential release events. (i) Macroscopically debris were collected on a clean polished metallic surface placed near the tester. (ii) Stat Peel’s respirable air samplers (Badges) were placed inside the tribometer chamber, while another air sampling badge was placed 2 m away outside the tribometer chamber (reference). The badges were run at the nominal flow rate of 91 mL/min for the duration of an abrasion experiment. (iii) An Apex2 Casella particle collector was used inside the tribometer chamber to collect particles onto a polycarbonate filter. The effective sampling time for particle collection was about 2 h. A scheme representing the experimental setup for the collection of abraded particles from the epoxy-TUBALL™ M207 sample is shown in Figure 2.

We would like to highlight that various studies have demonstrated the efficacy of carbon nanotubes in diminishing friction and wear. For instance, their application as a reinforcement phase in composites, protective films, solid lubricants, or lubricant additives has shown notable results. This reduction in friction is frequently attributed to the degradation of CNTs, which leads to the formation of a lubricating carbonaceous tribolayer, transition from graphitic-like to a more amorphous-like structures [21,22]. It is important to consider that during tribological test measurements, amorphous graphitic-like structures may be produced and deposited on the gridding ball’s surface. The quantity of these deposits is often not factored into the calculation of released CNTs captured by the particle collectors.

### 2.3. Particle Characterization Methods

Raman spectra were collected via a T-64000 (HORIBA Jobin Yvon, Vénissieux, France) micro-Raman system equipped with a 2D-CCD Symphony II detector. The excitation wavelength at 514.5 nm was provided by a DPSS laser (Cobolt Fandango TMISO laser, Norfolk, UK). The laser power on the sample was maintained at ~1 mW and focused on the samples by a microscope objective 50× (NA = 0.55). The collected scattered beam passed through an appropriate edge filter for the removal of the strong elastically scattered photons (LP02-514RU-25, Laser 2000, Cambridgeshire, UK) and was directed into the slit of the monochromator using the single spectrograph configuration. The resolution was kept constant in all experiments (≈6 cm^−1^). Instrumental calibration was performed via the standard 520.5 cm^−1^ Raman peak position of a Si wafer. This Raman system was used to analyze the surface of pristine and abraded samples, the collected macroscopic debris and airborne particles collected with the Apex2 Casella particle collector. 

An Identifier C2 system (Stat Peel, Glarus, Switzerland) was used for the quantification of respirable airborne SWCNTs. The system encompasses respirable air samplers (Badge, Stat Peel), filtration slides (Stat Peel) and a Raman spectrometer-based Reader (Stat Peel). The badge collects respirable airborne particles onto two 1-by-1 mm nanoporous membranes of the Stat Peel filtration slides, stages S and L. The sampling efficiency of the badges closely adopts the respirable convention as defined in ISO 7708 [23]. The filtration slides were analyzed by an Identifier C2 Reader. The reader uses a confocal Raman system for the quantification of materials collected on the filtration slides. A 785 nm laser was used for the excitation. The laser light was coupled into the Stat Peel optical pickup unit from the optical fiber via a collimator. The laser light was cleaned by a 785 nm laser line filter and then reflected into a 50× objective (NA = 0.75, WD = 1.6 mm) by a 785 nm dichroic filter and a broadband dielectric mirror. The laser spot size on the sample was ~20–25 µm, and the laser power (at sample position) was set to ~3.5 mW. The collected light follows the excitation path until the 785 nm dichroic filter, where the light components with longer wavelengths than 790 nm pass the dichroic filter and are further cleaned by a 785 nm dichroic long-pass filter. The collected light beam is coupled into an optical fiber by a fiber coupler, and the collection fiber is attached to a spectrograph equipped with a LDC-DD CCD camera. The wavelength calibration of the spectrometer was performed using the laser line and the 520 cm^−1^ Raman peak of silicon. 

The quantification of sampled SWCNT mass was performed by measuring the Raman spectrum of a 1.2-by-1.2 mm square (100 µm step size) positioned around the membrane. Each measured spectrum is fitted by the sum of a quadratic polynomial function (background) and appropriate number of Lorentzian line-shape functions. For the identification of SWCNTs, the fitted parameters of the line-shape functions are compared to reference values, and if the parameters are in their predefined acceptance range, then SWCNTs are considered to be identified in the given spectrum. The area of the Lorentzian functions of the identified spectra are summed up and normalized by the measured laser power. The obtained quantity is used for the quantification of SWCNT by comparing the value to the calibration curve of the given SWCNT. The reference samples of the calibration curve were prepared by the liquid deposition of reference dispersions of SWCNT using N-methyl-pyrrolidone as solvent. The residuals of N-methyl-pyrrolidone were removed by drying the membranes in a laboratory oven at 150 °C for at least 2 h. The airborne SWCNT exposures were calculated from the quantified masses and sampled air volumes.

## 3. Results

### 3.1. Characterization of Pristine, Abraded Samples and Debris

**Figure 3:**
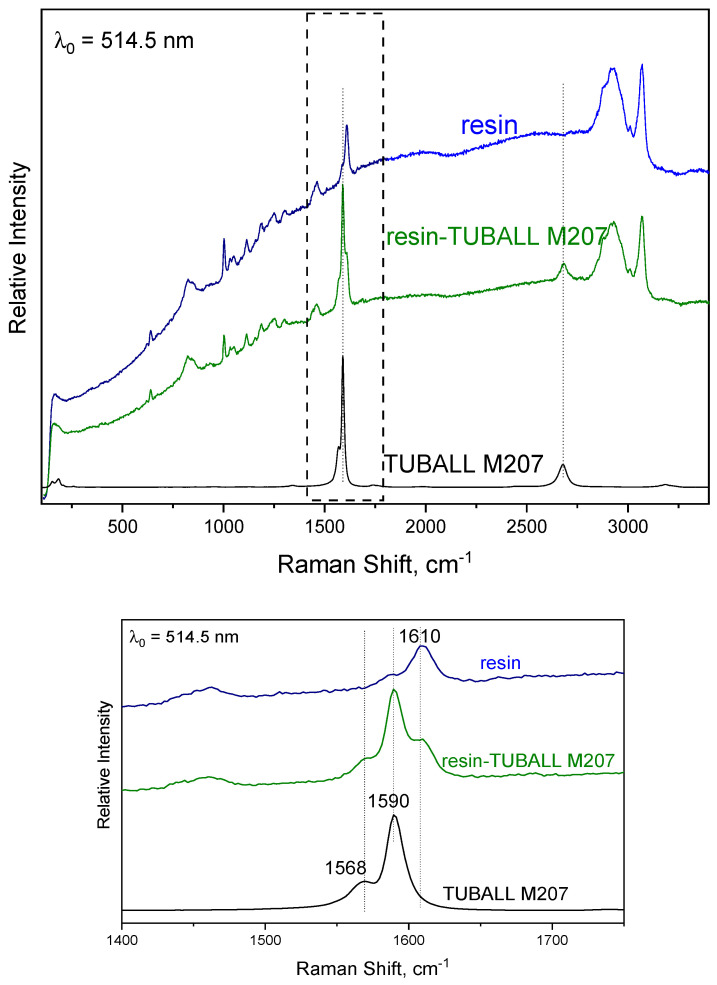
Raman spectra showing the characteristic bands attributed to SWCNTs (1590 cm^−1^) and to the epoxy resin (1610 cm^−1^): the whole spectral range (**top**) and centered at ~1600 cm^−1^, dashed square (**bottom**).

Neat samples as well as samples submitted to tribology tests and the debris collected were characterized by Raman spectroscopy, making use of the T-64000 micro-Raman system. In the case of abraded samples, the Raman spectra were obtained on the wear marks. The macroscopically produced debris by the abrasion of samples were collected on a clean polished metallic surface placed inside the tribometer. In Figure 3, the Raman spectra of TUBALL™ SWCNTs, the epoxy resin and the epoxy resin embedded with TUBALL™ Matrix207 are depicted. TUBALL™ SWCNT presents the characteristic G band at 1590 cm^−1^ together with a shoulder at 1570 cm^−1^, which is associated with vibrations of carbon atoms along the circumferential direction of the nanotube, while the 2D band appears at ~2700 cm^−1^ due to the second-order Raman scattering process [20,24,25]. The epoxy resin shows a peak at 1610 cm^−1^ corresponding to the phenyl groups, while the broad band at 2800–3000 cm^−1^ is due to the stretching CH_2_ groups in the aliphatic chain and the intense peak at 3070 cm^−1^ to the C-H stretching vibrations in an aromatic phenyl ring. In the Raman spectrum of the resin embedded with TUBALL™ Matrix 207, peaks corresponding to SWCNTs and epoxy are noticeable. The spectrum of the neat resin presents a shoulder at ~1590 cm^−1^; the relative intensities of the peaks at 1590 cm^−1^ (common to both epoxy resin and mainly to SWCNTs) and 1610 (only of epoxy resin) cm^−1^ are used as a probe for the presence of SWCNTs in the resin. Raman spectra of TUBALL™ SWCNTs and TUBALL™ Matrix 207 are depicted in Figure S1 (Supplementary Information).

**Figure 4:**
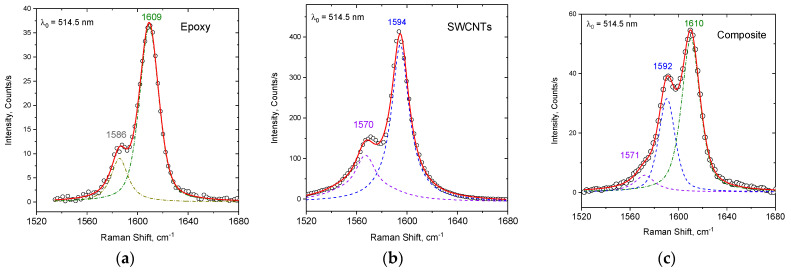
Representative fittings of the spectra centered at 1600 cm^−1^ with (**a**) two peaks for the epoxy sample, (**b**) two peaks for the SWCNTs sample and (**c**) three peaks for the composite. Dashed blue lines denote the individual Lorentzian peaks, solid red lines their addition while white circles correspond to original experimental data.

**Figure 5:**
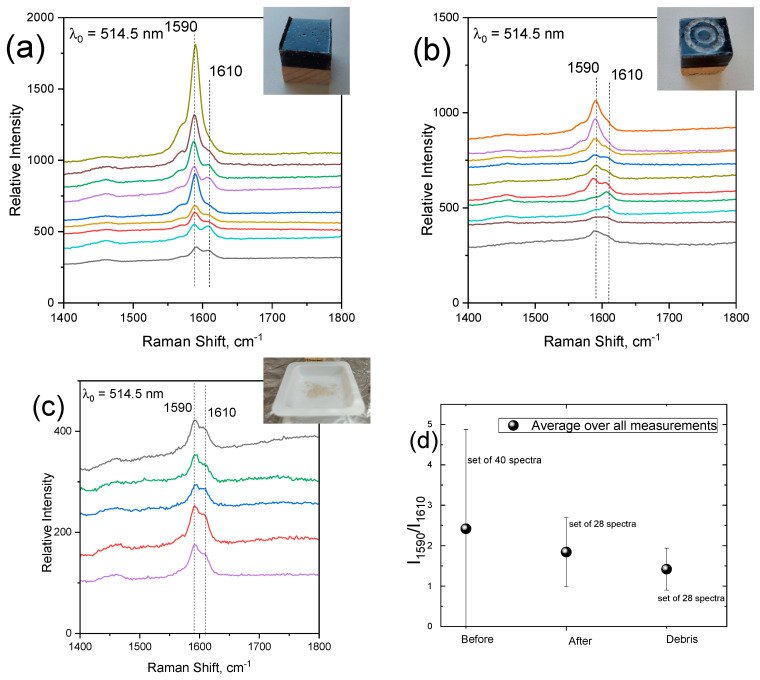
Raman spectra on several points on (**a**) resin-TUBALL™ Matrix 207 composite sample, (**b**) sample after abrasive wear (pin on disc) test, and (**c**) on several points on debris, indicating the peaks at 1590 cm^−1^ and 1610 cm^−1^ corresponding to SWCNT and epoxy, respectively. The pictures show the samples under study. (**d**) Statistics on the C = I_CNTs_/I_resin_ values for the composite before and after test for the collected debris.

Semi-quantitative results can be extracted by spectral decomposition of the contribution of each component by fitting the spectra of the pristine resin and TUBALL™-SWCNTs samples each with two peaks with Lorentzian line-shape functions (Figure 4). The composite sample should, in principle, be fitted with four peaks; however, since peaks of the two pristine materials at ~1590 cm^−1^ overlap, the fitting was performed using three peaks. The peak-integrated intensity ratio C = I_1590_/I_1610_ (C values) will thus be proportional to the SWCNT concentration in the composite at the excitation spot. In order to achieve more reliable results, the contribution of the resin’s peak at ~1590 cm^−1^ should be subtracted from the numerator; its integrated intensity value may be approximated as a fraction of the 1610 cm^−1^ band of the fitting, taking into account the relative intensity between 1610 and 1586 cm^−1^ (≈0.2) peaks for the pristine resin. Fittings were performed in the spectral region of interest for the case of 40 spectra obtained from different spots on the surface of untreated composite. Statistical analysis was conducted for the C values, yielding a result of C = 2.4 ± 2.5 for the untreated sample. The integrated intensity ratio values from the spectra obtained from the surface of the sample before tribology testing exhibit high inhomogeneity; the latter is straightforward by simple inspection of the variation of the intensity of the SWCNTs peak recorded from different spots on the sample (Figure 5a). The above-mentioned procedure associated with spectral decomposition was performed on spectra recorded from the same sample after the tribological tests (representative spectra are given in Figure 5b) as well as from the debris collected during the test (spectra are shown in Figure 5c). Statistics on the C values extracted for each case (number of spectra: 40 for the pristine sample, 26 for the abraded sample and 28 for the debris) are given in Figure 5d (black color datapoints). Despite the greater statistical sample offered by the number of accumulated spectra, the untreated sample is characterized by high error bars; in contrast to the other two sets of data associated with debris and the worn sample’s surface, the error bars are considerably smaller. Inspection of the C values for the pristine sample indicates that ~17% of the spectra exhibit C > 4. In contrast, a value C < 4 is obtained from all spectra recorded from the sample submitted to abrasive wear and for the debris (this explains the smaller error bars). The statistical analysis suggests that there is inhomogeneity (possibly aggregations, bundling, etc.) of the CNTs concentration on the surface of the untreated sample. The role of the dispersion of CNTs on the release of species after the application of tribological processes has been invoked [26]. There, it is argued that the number and size distribution of the released particles depends on the homogenous dispersion of CNTs; it was shown that the better the dispersion, the lower the number of particles released from the nanocomposite, which is certainly obvious. In our case, these highly SWCNTs-concentrated regions are absent on the newly formed surface after the abrasion test. They are also absent on the collected debris. It appears that the excess of CNTs (due to the aggregations) is “lost” after the abrasion tests. A possible explanation is that this excess may be released–transferred to the environment. This hypothesis motivated the use of particle collectors during the abrasive wear tests to further study the potential release of free SWCNTs or SWCNT-containing particles.

**Figure 6:**
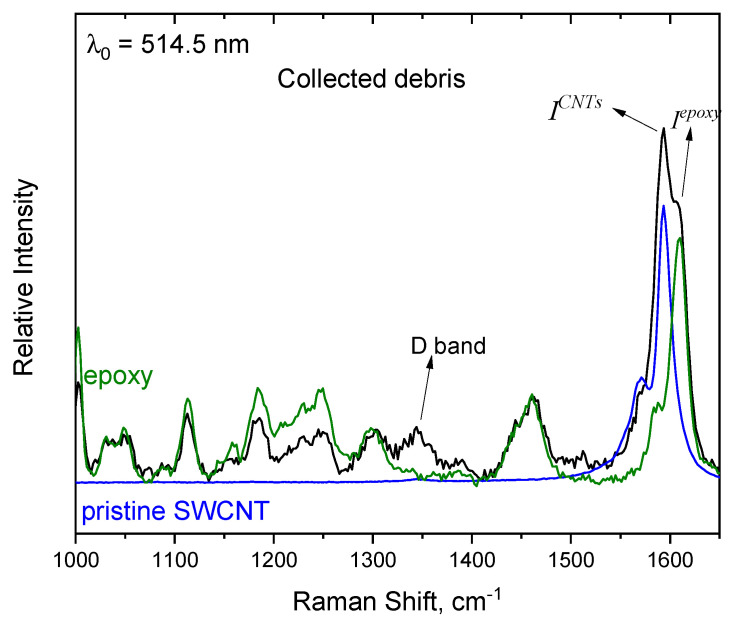
Comparison of SWCNT pristine spectrum (blue line), pristine resin–SWCNT (green line) spectrum and spectrum corresponding to collected debris (black line). The intensity of the D band in the latter spectrum indicates modifications caused by the tribological tests.

A careful examination of the spectra in the spectral region corresponding to the D-band of the SWCNTs, which is typically associated with structural defects (at ~1340 cm^−1^ when using the 514.5 nm excitation line or at ~1300 cm^−1^ when using the 785 nm excitation line [20,27]), reveals interesting observations. Prior to the abrasive wear test, the intensity of this D-band in our samples was relatively low, which is comparable to what is seen in the spectrum of pristine SWCNTs. However, after subjecting the samples to the abrasive wear test, a noticeable enhancement in the intensity of the D-band is observed in nearly all spectra obtained from the debris. To illustrate this, Figure 6 presents spectra for pristine TUBALL™ SWCNTs, a pristine resin-TUBALL™ Matrix 207 composite, and the spectrum recorded from the collected debris post-abrasive wear test. At ~1340 cm^−1^, the pristine resin spectrum exhibits no discernible bands, while the pristine SWCNTs spectrum shows a very faint band.

Intriguingly, in the spectrum of the collected debris, one can distinctly observe the contribution of the D-band. This observation suggests that the abrasion tests have induced structural modifications and the introduction of defects in the CNTs. Similar findings have been reported by other research groups [17,21,22], where Raman spectra obtained from samples containing carbon nanotubes (either SWCNTs or MWCNTs) submitted to ball-on-plate or pin-on-disk tests presented a broad peak at 1350 cm^−1^ or increase in this peak at the same time that I_G_/I_D_ decreased, indicating a formation of some amorphous carbon. These results may also support the transformation of the SWCNTs during friction. Since the peak at 1350 cm^−1^ corresponds to a Raman-active mode of a defective carbon network, the intensity is roughly proportional to the amount of amorphous carbon in the sample. 

We consider that during the tribology test, two scenarios may take place: an increase in structural/lattice defects or increase in disorder indicated by the increasing intensity of the D band that could be considered a degradation of carbon nanotubes and/or release of CNT-containing particles. To examine the 2^nd^ scenario, particle collectors were inserted in the tribometer chamber during the tests.

### 3.2. Particle Collector: STAT PEEL Identifier C2 System

Stat Peel’s Identifier C2 system was used to monitor the SWCNT content of respirable particles produced during the abrasion test. Two-membrane filtration slides were used with the air sampling badges that separate the aspirated respirable particles into two size fractions, which are then deposited onto two separate stages (membranes) S and L in the filtration slide. The lower limit of quantification (LoQ) for Tuball^TM^ SWCNT was estimated from the measurement of a linear standard calibration series, and it is approximately 5 pg for a single membrane.

Four respirable air samplers (Stat Peel badges with filtration slides #1, #2, #3 and #4) were placed inside the enclosure chamber of the tribometer during the abrasion test to collect respirable airborne particles produced by the tribometer. Additionally, one badge was placed 2 m away outside the tribometer (slide #5) to monitor exposure to CNTs at a certain distance to the emission source. We assume that all CNTs detected in these experiments originate from the abrasion of the sample. For Raman, since it is material-specific, it is irrelevant if other ambient particles are present in the sample, as they can be distinguished from the target material(s). SWCNT amounts were found above the LoQ on seven out of ten membranes of the five filtration slides that were used for sampling. The SWCNT amounts were below the LoQ on the stage L of slides #1, #2 and #4. The quantified mass and calculated exposure results are summarized in Table 1. All the quantified masses are close to the LoQ of the system, and the calculated exposures are three orders of magnitude lower than the NIOSH recommended exposure limit (REL) of 1 µg/m^3^ [28].

**Figure 7:**
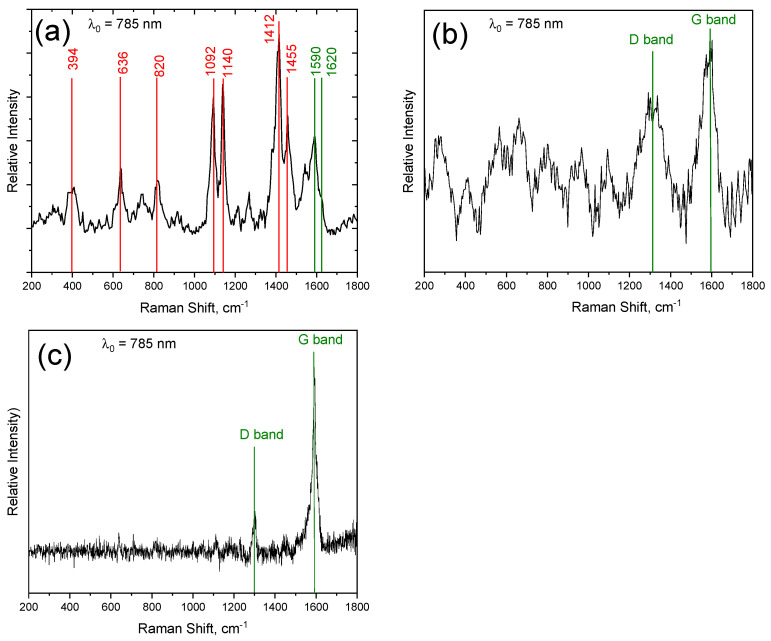
Raman spectra measured on the Stat Peel filtration membranes. (**a**) The green vertical lines at 1590 cm^−1^ and 1610 cm^−1^ denote the G band of TUBALL™ SWCNT and a characteristic Raman peak of the epoxy matrix, respectively. The red vertical lines depict unidentified Raman peaks. (**b**) The lower G/D band ratio might also indicate the degradation of SWCNTs during the abrasion test. (**c**) A small fraction of the measured spectra was observed with a high G/D band ratio.

A Stat Peel Reader unit with a confocal Raman system (785 nm laser) was used for the acquisition of the Raman spectra. A few measured spectra suggest that the dust produced by the tribometer contains embedded SWCNTs (see Figure 7a) where the Raman peak of SWCNT (G-band at ~1590 cm^−1^) and the Raman peak of the epoxy material at ~1610 cm^−1^ can be observed together, but mostly, the Raman spectra cannot be decomposed properly due to the low signal-to-noise ratios, as all detected masses are close to the LoQ of the method.

Furthermore, almost all the Raman spectra of SWCNTs found on the filtration slides exhibit a lower G-band height–D-band height ratio (G/D ratio) compared to the pristine Tuball^TM^ SWCNT (Figure 7b). The intensified D-band (located at ~1300 cm^−1^ when excited with a 785 nm laser) can refer to the degradation of the SWCNT during the abrasion test in accordance with the results of the collected debris [22]. Only a few recorded spectra were observed with higher G/D band ratios (Figure 7c) during the single particle analysis of the membranes performed with a Renishaw Invia Raman microscope equipped with a 785 nm laser. The corresponding light microscope image is shown in Figure S3 in the SI.

### 3.3. Particle Collector: Apex2 Casella

**Figure 8:**
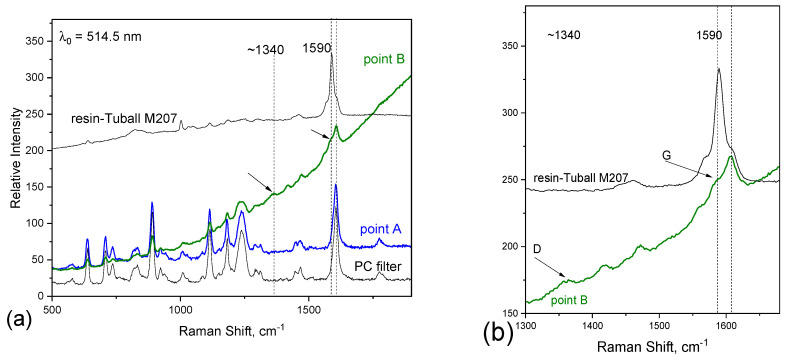
Raman spectra obtained from filter connected to Apex2 Casella. (**a**) On the bottom (black), the spectrum corresponding to the polycarbonate filter, in blue and green, the ones corresponding to random points on the filter and on top, the one corresponding to resin embedded with TUBALL™ Matrix 207. (**b**) Vibrational window where the bands corresponding to CNTs appear (indicated with arrows).

The Apex2 Casella particle collector was positioned inside the tribometer chamber during the abrasion wear tests, and examination of the filter by Raman spectroscopy (T64000 Raman spectrometer −514 nm) was performed. The filter material is polycarbonate, which presents a Raman peak at 1603 cm^−1^, which makes it difficult to elucidate between the filter, TUBALL™ SWCNT and epoxy resin. In Figure 8, the Raman spectra of the filter and the resin-TUBALL™ Matrix 207 composite together with spectra recorded on random points on the filter are depicted. From 20 points recorded on the filter, only one presented a Raman spectrum which indicated the presence of resin-TUBALL™ Matrix 207; all the others resemble the polycarbonate filter spectrum. This spectrum (the green one in Figure 8) shows a band at around 1340 cm^−1^ (with 514.5 nm excitation line), which corresponds to the D band in carbon nanotubes. Furthermore, it is noted that this band has increased compared to the resin-TUBALL™ Matrix 207 Raman spectrum, possibly indicating changes in the sample. The D band in the Raman spectrum of CNTs is associated with disorder-induced scattering, such as structural defects, impurities, or amorphous carbon content. An increase in the intensity of the D band suggests an increase in disorder or defects in the CNTs, indicating a degradation of the CNTS during tribology tests.

## 4. Conclusions

In this study, the potential release of single-walled carbon nanotubes (SWCNTs—brand TUBALL™) from epoxy composite materials was investigated. The evaluation of particle emissions was complemented by abrasive wear experiments conducted in a laboratory pin-on-disc tribometer setting with the goal of accelerating composite wear (which represent the worst case scenario), while the airborne particles were captured using particle collectors. 

On the μm scale (spatial resolution of μ-Raman spectroscopy), the inhomogeneity of the CNTs concentration in the epoxy matrix of pristine composite samples (possibly aggregations, bundling, etc.) was observed. Identification of free SWCNTs released from the epoxy matrix was identified in a series of Raman spectra performed on the Stat Peel filters; however, they were found to be scarce. A quantifiable amount of TUBALL™ carbon nanotubes was detected on all the Stat Peel filtration slides close to the limit of quantification of the method. However, the carbon nanotube exposures were approximately three orders of magnitude below the NIOSH REL of 1 µg/m^3^. 

Evidence of structural changes/degradation in SWCNTs in samples that underwent accelerated wear during abrasive tests were readily observed in the Raman spectra, as evidenced by the increased intensity of the characteristic band associated with defects in the nanotubes: the D-band. The increased number of defects corroborates the idea of SWCNTs rupturing during wear experiments.

Raman spectroscopy has been proved to be a crucial technique for the identification, characterization and quantification of SWCNTs released during abrasive wear tests on nanostructures composites. Furthermore, structural changes of the released nanomaterials can be assessed by Raman spectroscopy This information is valuable for industries and risk assessors, as it aids in evaluating the exposure potential of CNT-based products.

In conclusion, given the absence of highly concentrated SWCNT regions on the newly formed surface after the abrasion test, it can be argued that incorporating a finishing, gentle grinding process into the manufacturing of relevant industrial antistatic floors would enhance their safety by minimizing the potential release of nanomaterials in subsequent use.

## Figures and Tables

**Table 1:** Summary of TUBALL™ SWCNT mass and exposure results of the inspected filtration slides from field sampling. LoQ: limit of quantification (# denotes slide number).

Slide #	Respirable Mass, Stage S (ng)	Respirable Mass, Stage L (ng)	Respirable Exposure (µg/m^3^)
1	0.006	0.002 (<LoQ)	1.54 × 10^−3^
2	0.017	0.001 (<LoQ)	4.10 × 10^−3^
3	0.016	0.006	3.49 × 10^−3^
4	0.021	0.002 (<LoQ)	3.54 × 10^−3^
5	0.015	0.017	2.82 × 10^−3^

## Data Availability

The raw data will be available from corresponding author upon reasonable request.

## Supplementary Materials

The following supporting information can be downloaded at https://www.mdpi.com/article/10.3390/nano14010120/s1; Figure S1: Raman spectra showing the characteristic bands attributed to TUBALL™ SWCNT (top), TUBALL™ Matrix 207 (middle) and Araldite™ (bottom). Scanning electron microscopy. Figure S2. SEM images of stage S collection membrane #4 of the Stat Peel slides. Figure S3. Light microscope image of analyzed particle during single-particle analysis. Corresponding Raman spectrum is presented in Figure 7c in the main text.
