# Supplementary material for: Raman Spectroscopy Unfolds the Fate and Transformation of SWCNTs after Abrasive Wear of Epoxy Floor Coatings
Source: Nanomaterials (Basel). 2024 Jan 3;14(1):120. doi: 10.3390/nano14010120 (PMC10780583; doi:10.3390/nano14010120)
Supplement: Supplementary file 1 [file nanomaterials-14-00120-s001.zip › nanomaterials-2756415-supplementary.pdf]

Supplementary Material

Raman Spectroscopy Unfolds the Fate and Transformation of SWCNTs after Abrasive Wear of Epoxy Floor Coatings

Amaia Soto Beobide [1,*], Rudolf Bieri [2], Zoltán Szakács [2], Kevin Sparwasser [2,*], Ioanna G. Kaitsa [3], Ilias Georgiopoulos [4], Konstantinos S. Andrikopoulos [1,3], Gunther Van Kerckhove [5] and George A. Voyiatzis [1]

[1] Foundation for Research and Technology-Hellas (FORTH), Institute of Chemical Engineering Sciences (ICE-HT), Stadiou Str., 265 04 Rio-Patras, Greece; candrik@iceht.forth.gr (K.S.A.); gvog@iceht.forth.gr (G.A.V.)
[2] Stat Peel Ltd., Stampfgasse 4, CH-8750 Glarus, Switzerland; rudolf.bieri@statpeel.com (R.B.); zoltan.szakacs@statpeel.com (Z.S.)
[3] Department of Physics, University of Patras, 265 04 Rio-Patras, Greece; ioanna.kaitsa@protonmail.com
[4] MIRTEC S.A., Thiva Branch, 76th km of Athens-Lamia National Road, 320 09 Schimatari, Greece; i.georgiopoulos@mirtec.gr
[5] OCSiAl Europe Sarl, Rue de la Poudrerie 1, 3364 Leudelange, Luxembourg; gunther.van.kerckhove@ocsial.com
* Correspondence: asoto@iceht.forth.gr (A.S.B.); kevin.sparwasser@statpeel.com (K.S.)

Samples provided by OCSiAl:

TUBALL™ SWCNT: Single Wall Carbon Nanotubes (SWCNT), outer diameter 1.6 ± 0.4 nm.

TUBALL™ Matrix 207: Mix of Araldite (Oxirane, mono (C12-C14-alkyoxy methyl dereivs) and SWCNTs in a proportion of 90/10.

The Raman spectrum of TUBALL™-SWCNT sample is depicted in Figure S1. Two main features are observable, the graphite-like G band (~1580 cm^{-1}) and D band (~1350 cm^{-1}). The G band in the Raman spectrum of SWCNTs shows a doublet structure (split into G+ and G− components). The 2D band at ~2700 cm^{-1} is also appreciable. Besides G and D bands, another prominent feature at ~100–400 cm^{-1}, called the radial breathing mode (RBM), appears in the Raman spectrum of SWCNT. The frequency position of the RBM modes gives information about the distribution of single-walled carbon nanotube diameters according to the relation

$$\text{Diameter (nm)} = \frac{248}{RBM\ (cm^{-1})} \qquad (1)$$

It is in that sense that the diameter of TUBALL™-SWCNT sample from OCSiAL are in the 1.4-1.6 nm range.

Similar Raman spectrum is obtained under the same conditions for sample TUBALL™-Matrix 207 sample.

The main features observed in Araldite are the C-H stretching bands at 2800-3000 cm^{-1}, the CH_2 bending at ~1400 cm^{-1} and the characteristic epoxy ring band at 1260 cm^{-1}.

The Raman peaks belonging to Araldite are not discernible in the Raman spectrum of TUBALL™-Matrix 207 sample, the vibration bands of SWCNT are very active in Raman and overlap the oxirane peaks.

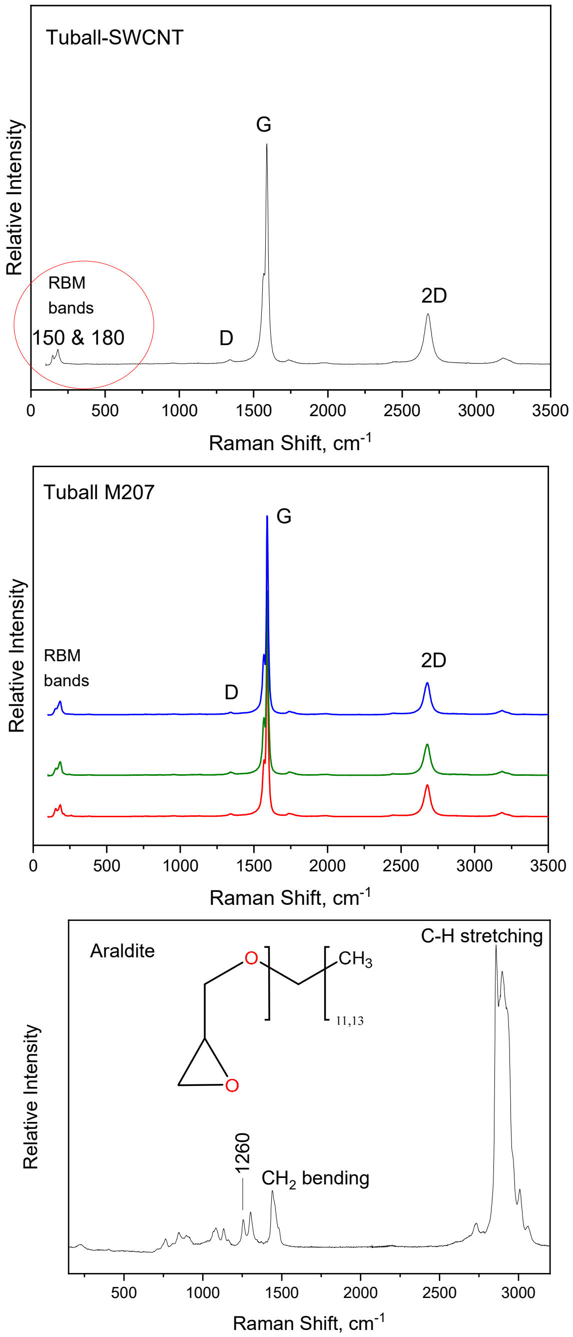

Figure S1. Raman spectra showing the characteristic bands attributed to TUBALL™ SWCNT (top), TUBALL™ M207 (middle) and Araldite (bottom).

Scanning electron microscopy

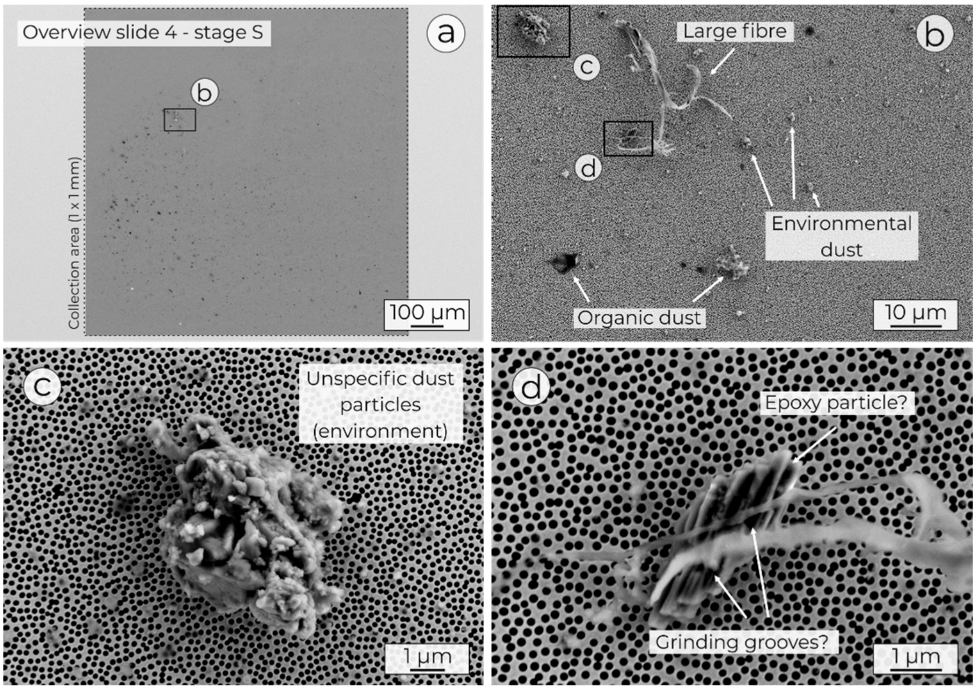

Figure S2. SEM images of stage S collection membrane #4 of the Stat Peel slides.

Filtration slides placed inside and outside the tribometer chamber were investigated with SEM. The inspection was aimed at identifying different particle morphologies, and possibly free or encapsulated CNTs on the collection membranes. The particle collection area (membrane) of the Stat Peel filtration slides is 1 mm^2. Time constraints rendered impossible the imaging of the entire membrane areas with magnification and resolution required to identify possible free or embedded CNTs. Regions of interest were defined based on obtained high-resolution overview images, in which selected particles were imaged in more detail. The scope of the analysis was limited to the identification of different particle morphologies, no further analytics, e.g. EDS was performed.

Figure S2 shows an example of the SEM imaging results obtained from Slide 4 (stage S). The most frequently found particle types on the slide inside the tribometer chamber include: unspecific dust agglomerates, organic depositions/dust particles identified by their instability under the electron beam in the SEM (fast degradation), large fibres (less common) likely from the environment (e.g. fabric fibre) (Fig. S2-ba). Longer fibres might be parts of a fabric of lab coat or similar. Note that the overall number of particles is low. The same particle types could also be identified on the slide that was outside the chamber; however, large fibres were observed more frequently. On both investigated slides, no free or embedded CNTs could be identified in the limited area inspected.

Scanning electron microscopy (SEM) imaging was performed with a Zeiss Merlin field emission gun equipped scanning electron microscope at the Scientific Center for Optical and Electron Microscopy at ETH Zürich. Secondary electron imaging was performed at an acceleration voltage of 5 kV and an approximate probe current of 100 pA. The inspection of SEM images was aimed at identifying different particle morphologies on the surface of the collection Stat Peel filtration slides.

Light microscope image

The spectrum shown in Fig. 7c in the main text was collected with a Renishaw Invia Raman microscope equipped with a 785 nm laser with an oval-shaped laser spot. The targeted particle and the approximate laser spot are shown in Fig. S3. Due to the relatively

large excitation area, it remains unclear if the CNT signal originated from the larger particle or from anywhere else in the excited area (CNT might well be not be visible with light microscope resolution).

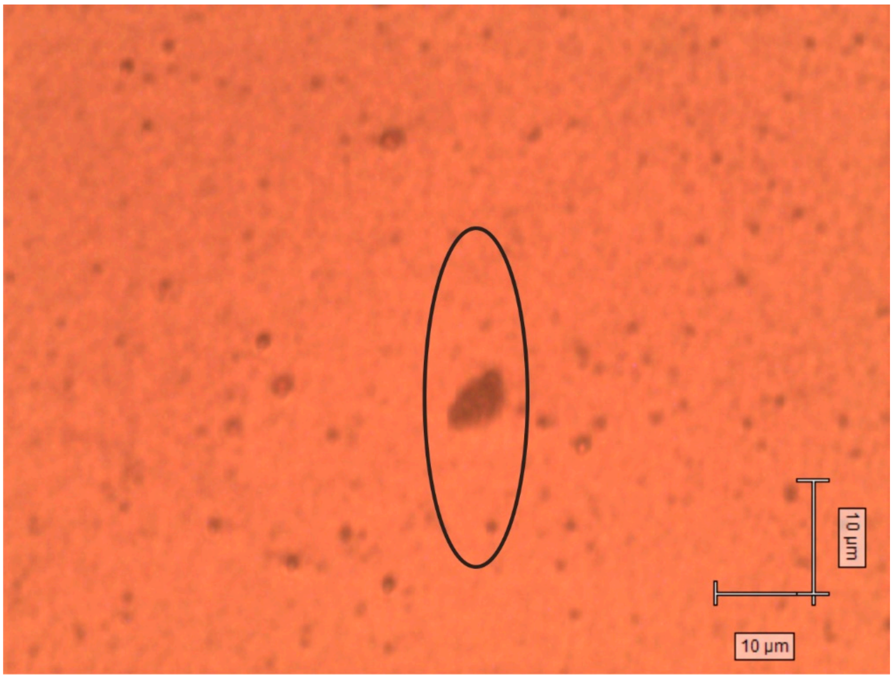

Figure S3. Light microscope image of analyzed particle during single-particle analysis. Corresponding Raman spectrum is presented in Fig. 7c in the main text.
